# Regulation of amino acid and nucleotide metabolism by crustacean hyperglycemic hormone in the muscle and hepatopancreas of the crayfish *Procambarus clarkia*

**DOI:** 10.1371/journal.pone.0221745

**Published:** 2019-12-26

**Authors:** Wenfeng Li, Kuo-Hsun Chiu, Chi-Ying Lee

**Affiliations:** 1 College of Ocean and Earth Sciences, Xiamen University, Fujian, China; 2 Department of Biology, National Changhua University of Education, Changhua, Taiwan; 3 Department of Aquaculture, National Kaohsiung University of Science and Technology, Kaohsiung, Taiwan; Technische Universität Dresden, GERMANY

## Abstract

To comprehensively characterize the metabolic roles of crustacean hyperglycemic hormone (CHH), metabolites in two CHH target tissues of the crayfish *Procambarus clarkii*, whose levels were significantly different between CHH knockdown and control (saline-treated) animals, were analyzed using bioinformatics tools provided by an on-line analysis suite (MetaboAnalyst). Analysis with Metabolic Pathway Analysis (MetPA) indicated that in the muscle Glyoxylate and dicarboxylate metabolism, Nicotinate and nicotinamide metabolism, Alanine, aspartate and glutamate metabolism, Pyruvate metabolism, and Nitrogen metabolism were significantly affected by silencing of CHH gene expression at 24 hours post injection (hpi), while only Nicotinate and nicotinamide metabolism remained significantly affected at 48 hpi. In the hepatopancreas, silencing of CHH gene expression significantly impacted, at 24 hpi, Pyruvate metabolism and Glycolysis or gluconeogenesis, and at 48 hpi, Glycine, serine and threonine metabolism. Moreover, analysis using Metabolite Set Enrichment Analysis (MSEA) showed that many metabolite sets were significantly affected in the muscle at 24hpi, including Ammonia recycling, Nicotinate and nicotinamide metabolism, Pyruvate metabolism, Purine metabolism, Warburg effect, Citric acid cycle, and metabolism of several amino acids, and at 48 hpi only Nicotinate and nicotinamide metabolism, Glycine and serine metabolism, and Ammonia recycling remained significantly affected. In the hepatopancreas, MSEA analysis showed that Fatty acid biosynthesis was significantly impacted at 24 hpi. Finally, in the muscle, levels of several amino acids decreased significantly, while those of 5 other amino acids or related compounds significantly increased in response to CHH gene silencing. Levels of metabolites related to nucleotide metabolism significantly decreased across the board at both time points. In the hepatopancreas, the effects were comparatively minor with only levels of thymine and urea being significantly decreased at 24 hpi. The combined results showed that the metabolic effects of silencing CHH gene expression were far more diverse than suggested by previous studies that emphasized on carbohydrate and energy metabolism. Based on the results, metabolic roles of CHH on the muscle and hepatopancreas are suggested: CHH promotes carbohydrate utilization in the hepatopancreas via stimulating glycolysis and lipolysis, while its stimulatory effect on nicotinate and nicotinamide metabolism plays a central role in coordinating metabolic activity in the muscle with diverse and wide-ranging consequences, including enhancing the fluxes of glycolysis, TCA cycle, and pentose phosphate pathway, leading to increased ATP supply and elevated protein and nucleic acid turnovers.

## 1. Introduction

Crustacean hyperglycemic hormone (CHH) is a polypeptide hormone originally identified in the X-organ/sinus gland (XO/SG) complex of the eyestalks [1–2]. Early biochemical studies of CHHs isolated from various decapod crustaceans showed they are of 72‒73 amino acid residues in length, with 3 disulfide bridges formed by 6 highly position-conserved cysteine residues [3–9]; peptide sequencing analyses indicated that CHH is N- and C-terminally blocked; the C-terminal amide was found critical for its hyperglycemic activity [10–14]. These and subsequent molecular studies [15–17] have established that CHH, being a prototypical member, belong to a family of polypeptide hormones, the CHH family, which also includes molt-inhibiting hormone (MIH), vitellogenesis-inhibiting hormone (VIH), and mandibular organ-inhibiting hormone (MOIH), and insect ion transport peptide (ITP) [18–21].

Combined data indicated that CHH is functionally involved with regulation of carbohydrate metabolism and responsible for mediating stress-induced hyperglycemia [22–27]. Specially, glycogen mobilization in two CHH target tissues (the hepatopancreas and muscle) has been shown to be stimulated by CHH (or tissue extracts containing CHH) through regulating activity of the enzymes involved in glycogen metabolism [28–32]; other studies suggested that CHH also stimulates glycolytic flux by increasing the availability of glycolysis substrate (glucose) [22, 33].

In the previous study of ours [34], a double-stranded RNA (dsRNA) technique was employed to effectively and specifically knock down CHH gene expression in the crayfish *Procambarus clarkii*. Metabolites in the tissue (the hepatopancreas and muscle) obtained from the CHH dsRNA-injected (DSI) and saline injected (SAI) animals were detected, identified, and quantified using a nuclear magnetic resonance (NMR)-based metabolomic method. Initial analysis of metabolites directly related to carbohydrate and energy metabolism suggested that CHH regulates tissue metabolism in tissue-specific and complementary manners [34]. In the present study, to fully characterize the effects of silencing CHH gene expression and understand the metabolic roles of CHH, we utilized web-based bioinformatics tools provided by MetaboAnalyst to comprehensively analyze all tissue metabolites whose levels changed significantly after CHH gene silencing. Results not only confirmed our previous conclusion that CHH regulates carbohydrate and energy metabolism but also extended the scope of CHH regulatory roles to amino acid and nucleotides metabolism. Finally, a complete profile of CHH metabolic function is presented and discussed.

## 2. Materials and methods

### 2.1 Ethics statement

All experiments using the crayfish (*P*. *clarkii*) were performed under the review committee of National Changhua University of Education (Permit number: NCUE-1052900151), in full accordance with the recommendations (Guidelines for Management and Use of Experimental Animals) set by the Council of Agriculture, Taiwan. Animals were purchased from local fishermen (Xihu River, Miaoli County, Taiwan), kept in freshwater tanks (water temperature: 24 ± 1°C; photoperiod: 12L:12D), and fed commercial shrimp food (Shanghai Inc., Taiwan) daily. Animals were anaesthetized with ice-cold water until immobile immediately before dissection.

### 2.2 Experimental animals and experimental procedures

Experimental treatment protocol, efficacy and specificity of gene silencing by CHH dsRNA, as well as NMR setting parameters, data acquisition and processing, and statistical analysis (Student's t-test) have been described in a previous study [34]. Briefly, double-stranded RNAs were produced using an *in vitro* transcription reaction driven by T7 promoter and T7 RNA Polymerase. Animals were treated with either saline injection (SAI) or CHH dsRNA injection (CHH DSI), and, at designated time points (24, 48, 72 hours after treatment), tissues were dissected under stereo-microscopes (SMZ745, Nikon) using fine scissors and used for a quantitative real-time PCR (the eyestalk ganglia) or a dual phase extraction procedure (the muscle and hepatopancreas). CHH dsRNA, but not green fluorescence protein dsRNA, effectively and significantly decreased CHH gene expression in the eyestalk ganglia at the time points examined. Aqueous phase extracts of the muscle and hepatopancreas were subjected to ^1^H Nuclear magnetic resonance (NMR) analysis (Varian Inova 500 MHz NMR spectrometer). The spectra were analyzed using a commercial software package (Chenomx NMR suite 4.6, Chenomx, Inc., Edmonton, Alberta, Canada) with the Chenomx 500-MHz (pH 6±8) library.

### 2.3 Metabolic Pathway Analysis (MetPA) and Metabolite Set Enrichment Analysis (MSEA)

Tissue metabolites whose levels were significantly different between the SAI and CHH DSI groups were subsequently analyzed using resources provided by a web-based tool suite MetaboAnalyst (v. 4.0, http://www.metaboanalyst.ca), specifically two functional analysis modules—Metabolic Pathway Analysis (MetPA) and Metabolite Set Enrichment Analysis (MSEA) [39, 40]. Both approaches work by comparing the significant compounds identified from the data uploaded by users to pre-defined functional groups [35].

MetPA combines statistical enrichment analysis with pathway topological characteristics to identify the most relevant pathways under the study condition [36, 37]. Briefly, the metabolites whose tissue levels were significantly different between SAI and CHH DSI groups were uploaded. After data processing and compound name mapping, pathway analysis was performed using the fruit fly (*Drosophila melanogaster*) library from the KEGG database [38]. The hypergeometric test was chosen to be pathway analysis algorithm for the over representation analysis and the relative betweenness centrality for pathway topology analysis.

The Metabolite Set Enrichment Analysis (MSEA) is a tool to test if there are some biologically meaningful groups (e.g., pathways) of metabolites that are significantly enriched. After data processing and compound name mapping, enrichment analysis was performed using the Overrepresentation Analysis (ORA) against a knowledge database, the Pathway-associated metabolite sets library, which contains 99 metabolite sets based on normal metabolic pathways, in the Small Molecular Pathway Database (SMPDB) [39].

## 3. Results

Levels of 149 and 181 metabolites in the muscle at 24 and 48 hpi (hour post injection), respectively, and those of 24 and 12 metabolites in the hepatopancreas at 24 and 48 hpi, respectively, were found significantly different between the saline-injected (SAI) and CHH double-stranded RNA-injected (CHH DSI) animals (*Procambarus clarkii*). To give a comprehensive survey of the effects of silencing CHH gene expression on tissue metabolism, all significantly changed metabolites were analyzed using MetPA (Metabolic Pathway Analysis) and MSEA (Metabolite Set Enrichment Analysis) with web-based resource (MetaboAnalyst, v. 4.0).

### 3.1 MetPA

Analysis with MetPA indicated that the following pathways were significantly affected by silencing of CHH gene expression at 24 hpi in the muscle (Table 1 and S1 Table): Glyoxylate and dicarboxylate metabolism, Nicotinate and nicotinamide metabolism, Alanine, aspartate and glutamate metabolism, Pyruvate metabolism, and Nitrogen metabolism. At 48 hpi, only Nicotinate and nicotinamide metabolism remained significantly affected (Table 1 and S1 Table).

**Table 1:** KEGG metabolic pathways significantly impacted in the muscle of the crayfish *Procambarus clarkii* by silencing of CHH gene expression as revealed by Metabolic Pathway Analysis.

Time point	Pathway	Total^a^	Hits^b^	*p*-Value^c^
24 hpi	Glyoxylate and dicarboxylate metabolism	16	7	0.0019
	Nicotinate and nicotinamide metabolism	9	5	0.0025
	Alanine, aspartate and glutamate metabolism	23	7	0.019
	Pyruvate metabolism	24	7	0.024
	Nitrogen metabolism	7	3	0.047
48 hpi	Nicotinate and nicotinamide metabolism	9	5	0.005

^**a**^Total genes in the biological pathway.

^**b**^Number of genes hit in the biological pathway.

^**c**^*p*-Value calculated from raw data.

In the hepatopancreas, silencing of CHH gene expression significantly impacted Pyruvate metabolism and Glycolysis or gluconeogenesis at 24 hpi, and Glycine, serine and threonine metabolism at 48 hpi (Table 2 and S2 Table).

**Table 2:** KEGG metabolic pathways significantly impacted in the hepatopancreas of the crayfish *Procambarus clarkii* by silencing of CHH gene expression as revealed by Metabolic Pathway Analysis.

Time point	Pathway	Total^a^	Hits^b^	*p*-Value^c^
24 hpi	Pyruvate metabolism	24	3	0.010
	Glycolysis or Gluconeogenesis	25	3	0.011
48 hpi	Glycine, serine and threonine metabolism	25	2	0.019

^**a**^Total genes in the biological pathway.

^**b**^Number of genes hit in the biological pathway.

^**c**^*p*-Value calculated from raw data.

### 3.2 MSEA

Analysis using MSEA showed that several Small Molecular Pathway Database (SMPDB) metabolite sets were significantly affected in the muscle at 24 hpi (Table 3 and S3 Table), including Ammonia recycling, Nicotinate and nicotinamide metabolism, Aspartate metabolism, Pyruvate metabolism, Alanine metabolism, Glutamate metabolism, Purine metabolism, Arginine and Proline metabolism, Glycine and serine metabolism, Warburg effect, and Citric acid cycle (Table 3 and S3 Table). At 48 hpi, the metabolic groups that remained significantly affected included Nicotinate and nicotinamide metabolism, Glycine and serine metabolism, and Ammonia recycling (Table 3 and S3 Table).

**Table 3:** SMPDB metabolite sets significantly impacted in the muscle of the crayfish *Procambarus clarkii* by silencing of CHH gene expression as revealed by Metabolite Set Enrichment Analysis.

Time point	Pathway	Total^a^	Hits^b^	*p*-Value^c^
24 hpi	Ammonia Recycling	32	13	0.0002
	Nicotinate and Nicotinamide Metabolism	37	14	0.0003
	Aspartate Metabolism	35	12	0.002
	Pyruvate Metabolism	48	14	0.005
	Alanine Metabolism	17	7	0.006
	Glutamate Metabolism	49	14	0.006
	Purine Metabolism	74	18	0.012
	Arginine and Proline Metabolism	53	14	0.013
	Glycine and Serine Metabolism	59	15	0.015
	Warburg Effect	58	14	0.029
	Citric acid cycle	32	9	0.03
48 hpi	Nicotinate and Nicotinamide Metabolism	37	14	0.002
	Glycine and Serine Metabolism	59	19	0.003
	Ammonia Recycling	32	12	0.005

^**a**^Total genes in the biological pathway.

^**b**^Number of genes hit in the biological pathway.

^**c**^*p*-Value calculated from raw data.

In the hepatopancreas, MSEA analysis showed that Fatty acid biosynthesis was significantly impacted at 24 hpi (Table 4 and S4 Table).

**Table 4:** SMPDB metabolite sets significantly impacted in the hepatopancreas of the crayfish *Procambarus clarkii* by silencing of CHH gene expression as revealed by Metabolite Set Enrichment Analysis.

Time point	Pathway	Total^a^	Hits^b^	*p*-Value^c^
24 hpi	Fatty Acid Biosynthesis	35	3	0.045

^**a**^Total genes in the biological pathway.

^**b**^Number of genes hit in the biological pathway.

^**c**^*p*-Value calculated from raw data.

### 3.3 Changes in amino acid and nucleotide metabolic profiles

Combined results derived from analyses of MetPA and MSEA indicated that silencing of CHH gene expression significantly affected metabolism of carbohydrates (in the muscle and hepatopancreas), nucleotides and amino acids (mainly in the muscle), and fatty acids (in the hepatopancreas). A comprehensive discussion on carbohydrate and energy metabolism in the muscle and hepatopancreas has been reported previously [34]. Here, changes in the profile of metabolites in amino acid and nucleotide metabolism will be described in details.

**Fig 1:**
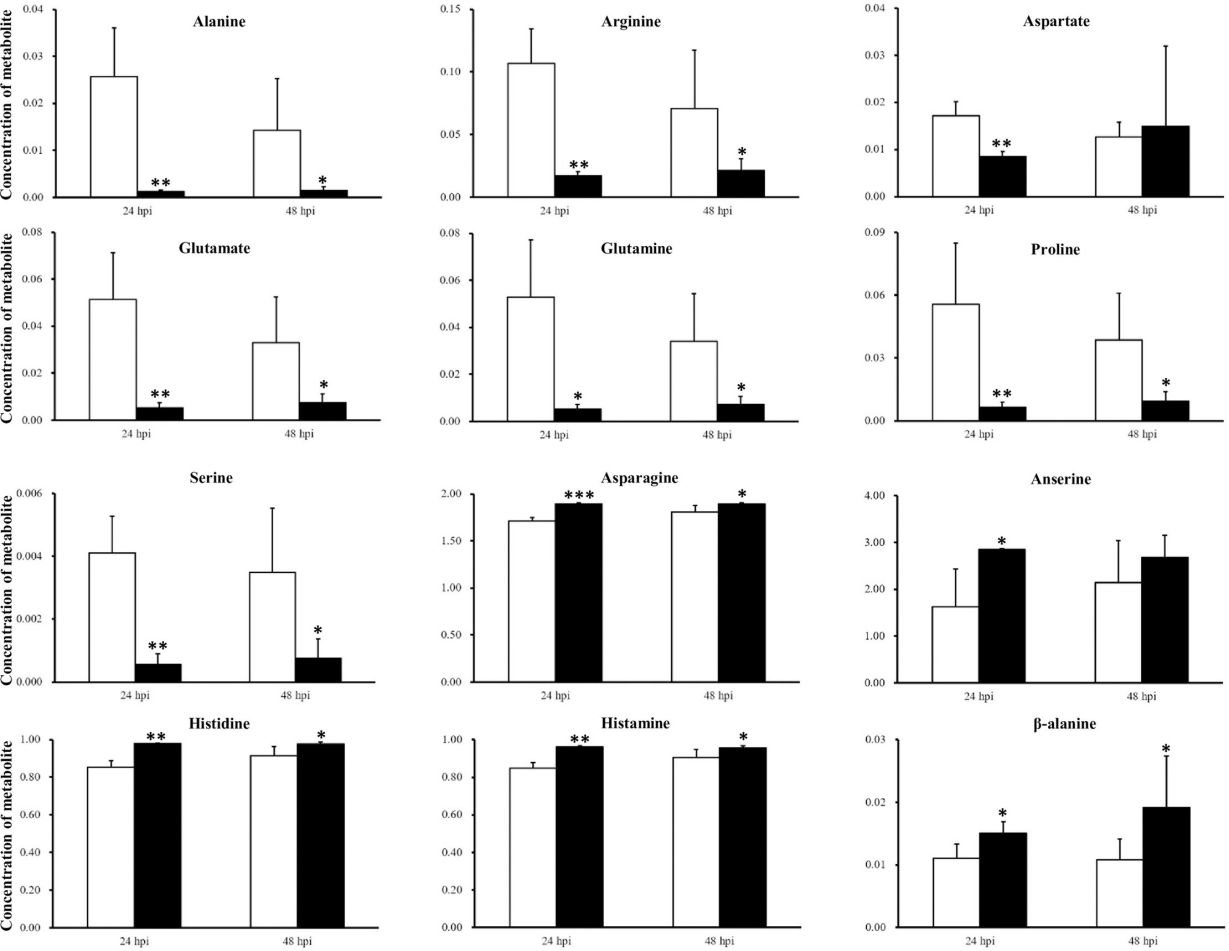
Amino acids and related compounds whose concentration significantly changed by silencing of CHH gene expression in the muscle of the crayfish *Procambarus clarkii* at 24 and 48 hpi. White bar, saline-injected group (SAI); black bar, CHH double-stranded RNA-injected group (CHH DSI). *, p<0.05; **, p<0.01; ***, p<0.001.

In the muscle at 24 hpi, levels of several amino acids, including alanine, arginine, aspartate, glutamate, glutamine, proline and serine decreased significantly, while those of 5 amino acids or related compounds, asparagine, anserine, histidine, histamine, and β-alanine, significantly increased (Fig 1). At 48hpi, the changes were similar to those at 24 hpi, with aspartate no longer significantly decreased and anserine no longer significantly increased (Fig 1).

**Fig 2:**
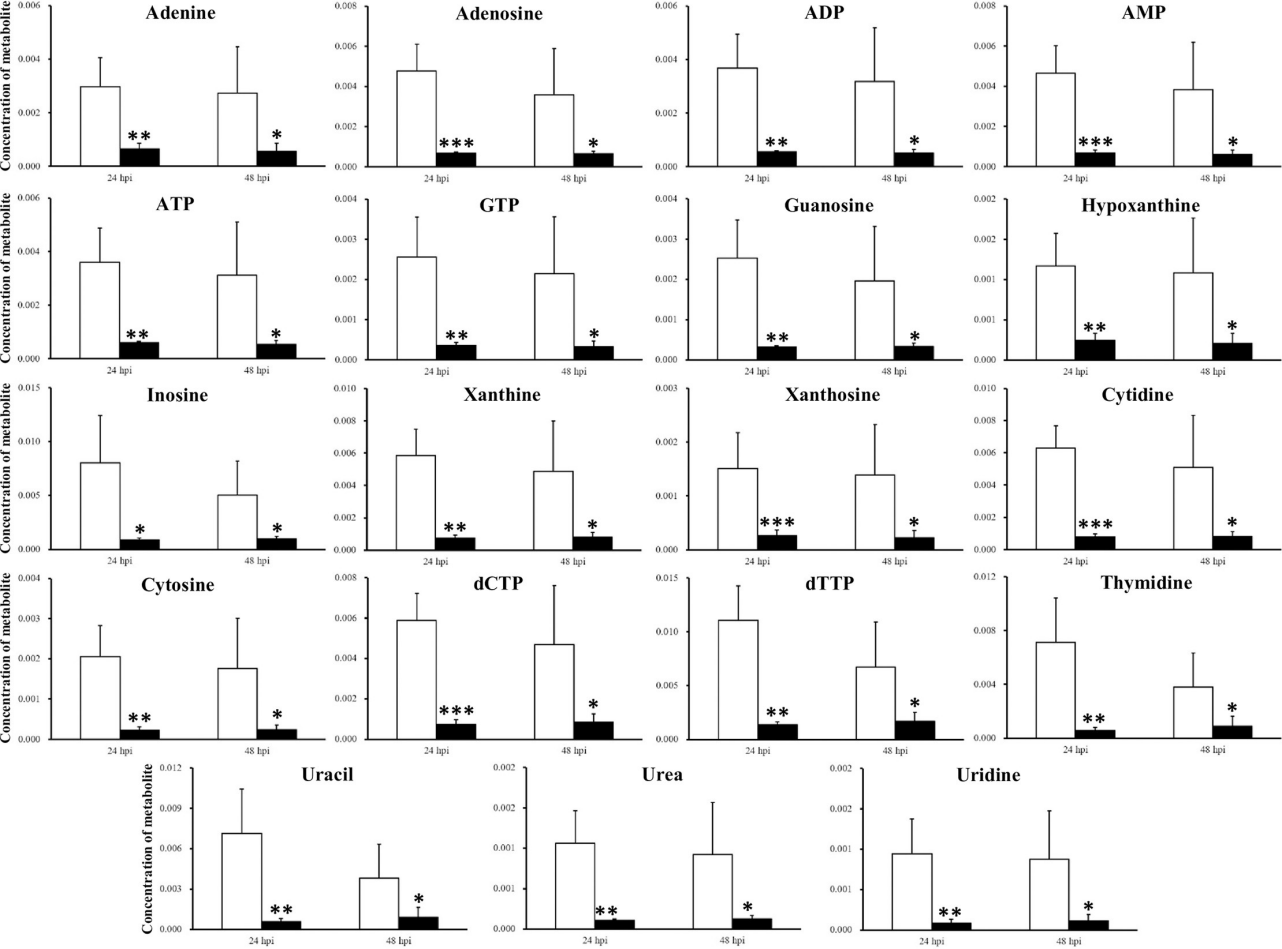
Metabolites involved in nucleotide metabolism whose concentration significantly changed by silencing of CHH gene expression in the muscle of the crayfish *Procambarus clarkii*. White bar, saline-injected group (SAI); black bar, CHH double-stranded RNA-injected group (CHH DSI). *, p<0.05; **, p<0.01; ***, p<0.001.

In response to silencing of CHH gene expression in the muscle at 24 hpi, metabolite levels involved in nucleotide metabolism significantly decreased almost across the board, including adenine, adenosine, ADP, AMP, ATP, GTP, guanosine, hypoxanthine, inosine, xanthine, xanthosine, cytidine, cytosine, dCTP, dTTP, thymidine, uracil, urea and uridine (Fig 2). Similar trends of changes were observed at 48 hpi (Fig 2).

In the hepatopancreas, with regard to nucleotide metabolism only levels of thymine and urea were significantly decreased at 24 hpi and no significant change was found at 48 hpi.

## 4. Discussion

In the present study, comprehensive analysis of the metabolite whose levels were significantly changed by silencing CHH gene expression revealed that the effects were not limited to carbohydrate and energy metabolism as previous studies had indicated [22, 33, 34]. Analysis of the metabolite whose levels were significantly changed by silencing CHH gene expression revealed that the effects were not limited to carbohydrate and energy metabolism as previous studies had indicated. Thus, combined data derived from MetPA and MSEA revealed additionally that, in the muscle nucleotide metabolism was severely reduced, likely due to a negatively impacted pentose phosphate pathway (PPP), and amino acid metabolism significantly affected that would likely result in slowing down protein synthesis, while in the hepatopancreas fatty acid biosynthesis was significantly affected.

**Fig 3:**
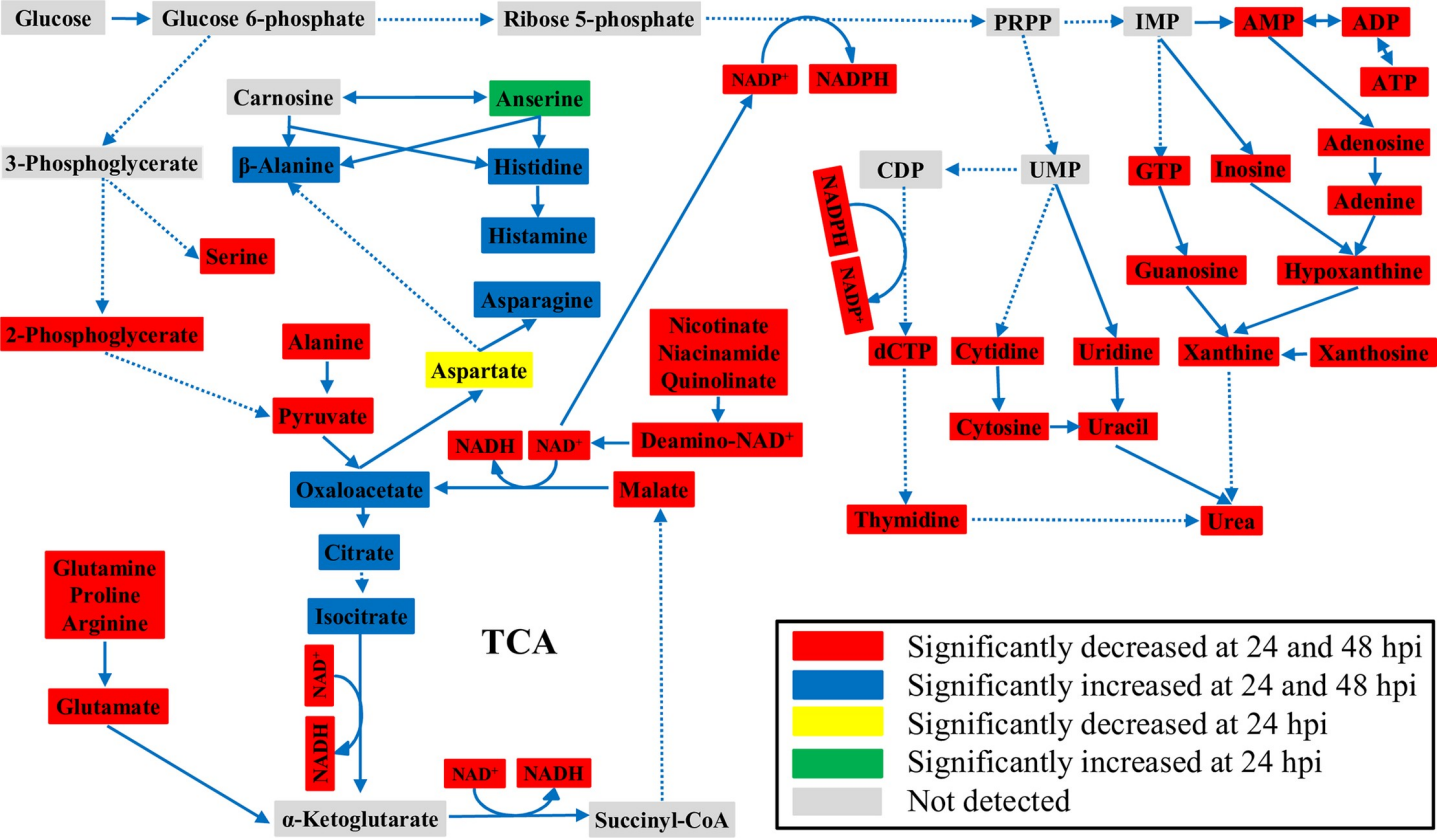
Metabolic profiles of the muscle of the crayfish *Procambarus clarkii* after CHH dsRNA treatment. Red rectangle: significantly decreased from saline-injected levels at both 24 and 48 hpi; blue rectangle: significantly increased from saline-injected levels at both 24 and 48 hpi; yellow rectangle: significantly decreased from saline-injected levels only at 24 hpi; green rectangle: significantly increased from saline-injected levels only at 24 hpi; gray rectangle: not detected. Dotted lines indicate multiple metabolic steps are involved that are not individually specified. ADP: adenosine diphosphate, AMP: adenosine monophosphate, ATP: adenosine triphosphate, CDP: cytidine diphosphate, dCTP: deoxycytidine triphosphate, Glucose 6-P: glucose 6-phosphate, GTP: guanosine triphosphate, IMP: inosine monophosphate, NAD^+^/ NADH: oxidized/reduced nicotinamide adenine dinucleotide, NADP^+^/NADPH: oxidized/reduced nicotinamide adenine dinucleotide phosphate, Ribose 5-P: ribose 5-phosphate; PRPP: phosphoribosyl pyrophosphate, UMP: uridine monophosphate.

Specifically, in the muscle, one of the pathways significantly negatively affected at both 24 hpi and 48 hpi is Nicotinate and nicotinamide metabolism, which concerns the metabolism of two nicotinamide coenzymes (NAD^+^ and NADP^+^) and has metabolically diverse ramification, with levels of both coenzymes being significantly reduced (Table 1 and S1 Table). We have suggested that low availability of NAD^+^ was central to the metabolic changes in the muscle in response to silenced CHH gene expression, which led to reduced fluxes of glycolysis and TCA cycle and eventually to diminished ATP levels [34]. In the same vein, low availability of NADP^+^ could reduce the flux of pentose phosphate pathway (PPP), resulting in lower levels of two PPP metabolites, NADPH and ribose 5-phosphate, which have diverse metabolic functions [41, 42]. NADPH is consumed in reductive reactions including the reduction of ribonucleotides to deoxyribonucleotides, whereas ribose 5-phosphate, via PRPP (5-phosphoribosyl 1-pyrophosphate), is a precursor to nucleotide biosynthesis [43] (see Fig 3). Thus, a negatively impacted PPP would, as a result of low NADP^+^ levels, decrease nucleotide biosynthesis, which was clearly demonstrated by significantly lower levels of nucleotides across the board (Figs 2 and 3).

Another pathway that was significantly affected in the muscle by CHH gene silencing at 24 hpi is Pyruvate metabolism (Table 1 and S1 Table). Pyruvate sits at a site where several metabolic pathways intersect. A significantly affected pyruvate metabolism is compatible with the observations that glycolysis and TCA cycle were negatively impacted in the muscle when CHH gene expression was silenced [34]. Pyruvate could also be used to directly form alanine or converted via a TCA intermediate (α-ketoglutarate) to other non-essential amino acids such as glutamate, glutamine, arginine, proline [44], whose levels were all decreased after CHH gene expression was silenced (Figs 1 and 3). Moreover, a glycolytic intermediate (3-phosphoglycerate) could serve as a precursor for the biosynthesis of serine [44], levels of which were significantly decreased (Figs 1 and 3). Indeed, analyses with MetPA and MSEA confirmed that Alanine, aspartate and glutamate metabolism and several amino acid metabolite sets (Aspartate metabolism, Alanine metabolism, Glutamate metabolism, Arginine and proline metabolism, Glycine and serine metabolism) was negatively affected (Tables 1 and 3, S1 and S3 Tables). On the other hand, levels of several amino acids and related metabolites in the muscle were increased after silencing CHH gene expression; these included those of asparagine, anserine, histidine, histamine, and β-alanine (Fig 1). Aspartate and asparagine are two of the amino acids that could be synthesized from a TCA intermediate (oxaloacetate) [44], whose levels was shown to be significantly accumulated because of inhibition of the 3 NAD^+^-dependent TCA reactions that carry isocitrate through to oxaloacetate due to low NAD^+^ levels [34]. Thus, an increase in asparagine levels was likely a spillover from a TCA cycle thus inhibited (Fig 3). In addition, increases in β-alanine, histidine, and anserine levels (Fig 1) are worth mentioning. Carnosine (not detected) is a dipeptide, highly concentrated in the muscle and brain, consists of β-alanine and histidine, and could be converted to anserine via methylation of the histidine residue (Fig 3) [45]. Carnosine has been shown to be able to, among others, scavenge and detoxify reactive oxygen species and reactive aldehyde derived from lipid peroxidation [45, 46]. Thus, elevated levels of β-alanine, histidine, and anserine suggest that degradation (to β-alanine and histidine) and conversion (to anserine) from carnosine were enhanced under silenced CHH gene expression and imply that CHH has protective roles for the muscle cells by increasing carnosine levels via combination of β-alanine and histidine and conversion from anserine. Finally, Glyoxylate and dicarboxylate metabolism, which was highly significantly impacted by silenced CHH gene expression (see Table 1 and S1 Table), describes a variety of reactions involving glyoxylate or dicarboxylates that interconnect with several aspects of cellular metabolism, including Pyruvate metabolism and TCA cycle, Glycine, serine and threonine metabolism, Purine metabolism, Nitrogen metabolism that were also impacted as revealed by MetPA and MSEA.

In the hepatopancreas, the effects of silencing CHH gene expression were comparatively milder than in the muscle. Significantly affected pathways include Pyruvate metabolism and Glycolysis or Gluconeogenesis, which are consistent with our previous suggestion that glycolysis was inhibited and gluconeogenesis stimulated [34].

MSEA analysis of the metabolites, other than confirmed affected pathways as suggested by MetPA, provided additional insights regarding the effects of silencing CHH gene expression. Thus, in the muscle, MSEA analysis identified Warburg Effect as a metabolite set being significantly negatively affected in response to CHH gene silencing at 24 hpi (see Table 3 and S3 Table). An invertebrate Warburg effect, characterized by up-regulation of several metabolic pathways, including glycolysis, the pentose phosphate pathway, ribonucleotide biosynthesis, glutaminolysis and amino acid biosynthesis, was observed in the tissues of the white spot syndrome virus (WSSV)-infected shrimps [47]. Based on the tissue responses (in the muscle) to silencing of CHH gene expression, the metabolic profile of the Warburg effect induced by WSSV infection is consistent with what would be expected when CHH release is greatly enhanced. Indeed, infection of white spot syndrome virus enhanced a rapid and long-lasting release of CHH into hemolymph [48]. Thus, the combined data support the notion that the WSSV-induced Warburg effect is at least in part due to the metabolic effects of CHH released by WSSV infection. In addition, MSEA also revealed that in the muscle Ammonia recycling, which involves deamination of excess amino acids and purine nucleotides [44], was negatively impacted (see Table 3 and S3 Table), which, combined with results showing significantly lower levels of amino acids and nucleotides (Figs 1 and 2), indicated a metabolic status of low protein and nucleic acid turnovers. Moreover, analysis with MSEA indicated that in the hepatopancreas fatty acid biosynthesis was significantly affected (Table 4 and and S4 Table). Due to the limitation of the methodology employed by the present study, only water soluble short-chained fatty acids (acetate, butyrate, caprate) were detected, whose levels decreased significantly (S4 Table). The results obtained under silenced CHH gene expression, though only a small sub-set of fatty acids were detected and quantified, are consistent with those from a previous study showing that CHH stimulated lipid mobilization from the storage tissues (*e*.*g*., the hepatopancreas), releasing free fatty acids into hemolymph, to be used metabolically in the consuming tissues [49].

## 5. Conclusion

**Fig 4:**
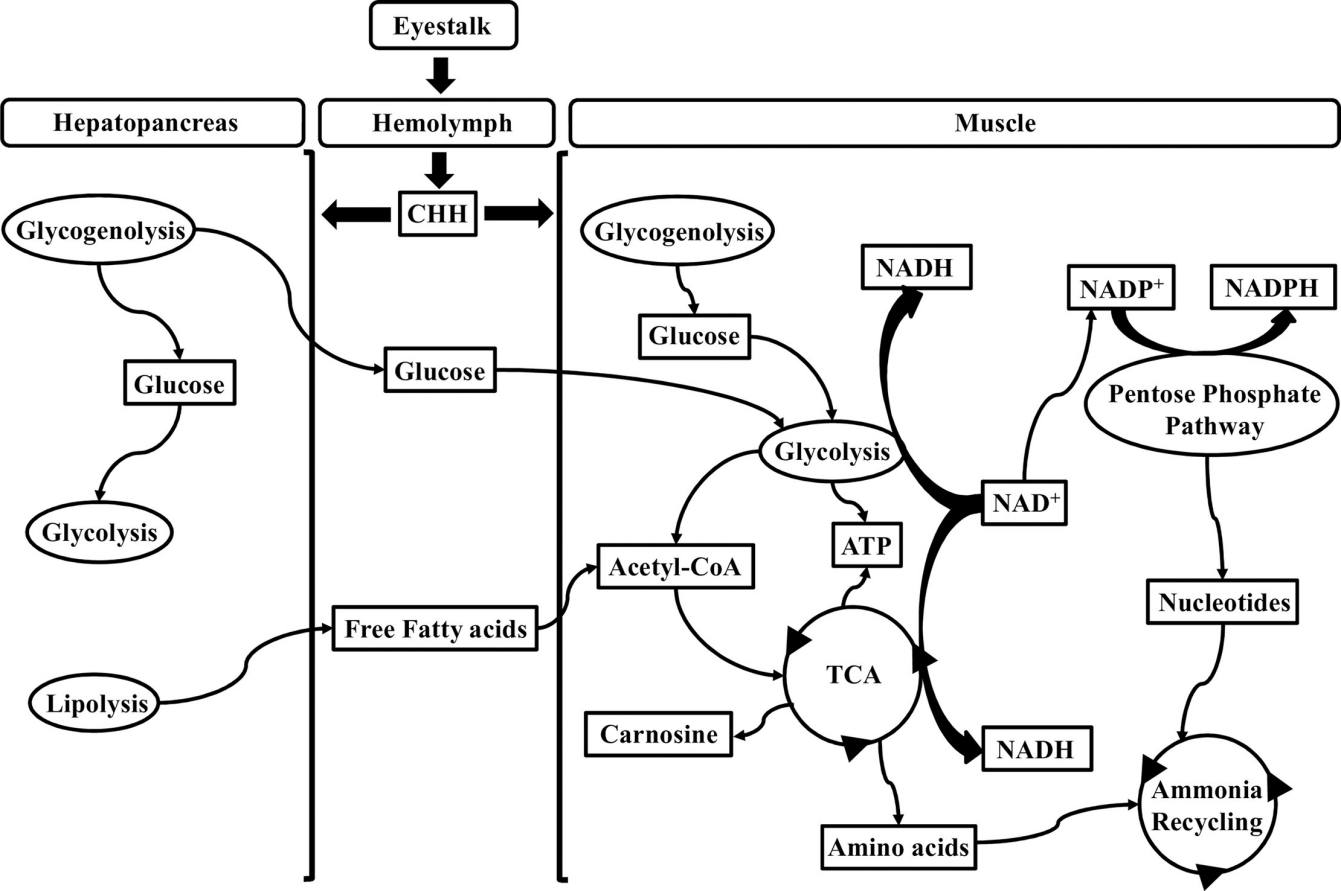
Proposed metabolic roles of CHH in the muscle and hepatopancreas.

In summary, the present study, combined with methods developed in a previous study [34], established a metabolomics pipeline that includes an NMR-based detection and identification of tissue metabolites and a web-based tool suite MetaboAnalyst allowing interpretation of metabolomics data in the context of metabolic network. In conjunction with a dsRNA-based gene silencing method [34], the metabolomics pipeline characterized the metabolic effect of CHH on its target tissues as far more diverse than previously realized. In brief, CHH clearly has differential effects on the 2 target tissues, with the muscle much more heavily regulated by CHH. Whereas CHH is expected to stimulate glycolysis and lipolysis in the hepatopancreas, its stimulatory effect on nicotinate and nicotinamide metabolism plays an important role in the muscle coordinating stimulation of the fluxes of glycolysis, TCA cycle, and pentose phosphate pathway, leading to increased ATP supply and elevated protein and nucleic acid turnovers. Combining our data and previous ones [22, 28, 29, 31, 33, 34, 49, 50], a comprehensive scheme of CHH roles in the 2 target tissues is proposed (Fig 4). Under conditions when its release from the eyestalk ganglia into the hemolymph is stimulated [23, 24, 25, 26, 27, 48], CHH acts on and differentially regulates its target tissues. Hence, while CHH stimulates glycogenolysis in both target tissues, resulting in higher levels of glucose [28, 29, 30, 31, 50] that drives glycolytic flux [22, 33], in the hepatopancreas, CHH additionally enhances lipolysis [49 and the present study]. Glucose and free fatty acids are released into the hemolymph and taken up by the muscle where they are further metabolized via glycolysis and TCA cycle, respectively, for ATP production. In the muscle, central to the effects of CHH is a stimulated Nicotinate and nicotinamide metabolism, which provides two nicotinamide coenzymes (NAD^+^ and NADP^+^) that drive glycolysis and TCA cycle, and the pentose phosphate pathway, respectively [34 and the present study], resulting in more ATP supply and higher protein and nucleic acid turnover. Additionally, CHH may provide protective effects to the muscle by increasing carnosine levels.

## Supporting information

S1 Table Effects of CHH dsRNA treatment on levels of metabolites involved in the KEGG metabolic pathways significantly impacted by silencing of CHH gene expression in the muscle of the crayfish *Procambarus clarkii*. (PDF)

S2 Table Effects of CHH dsRNA treatment on levels of metabolites involved in the KEGG metabolic pathways significantly impacted by silencing of CHH gene expression in the hepatopancreas of the crayfish *Procambarus clarkii*. (PDF)

S3 Table Effects of CHH dsRNA treatment on levels of metabolites involved in the SMPDB metabolite sets significantly impacted by silencing of CHH gene expression in the muscle of the crayfish *Procambarus clarkii*. (PDF)

S4 Table Effects of CHH dsRNA treatment on levels of metabolites involved in the SMPDB metabolite sets significantly impacted by silencing of CHH gene expression in the hepatopancreas of the crayfish *Procambarus clarkii*. (PDF)
