# Supplementary material for: Regulation of amino acid and nucleotide metabolism by crustacean hyperglycemic hormone in the muscle and hepatopancreas of the crayfish Procambarus clarkia
Source: PLoS One. 2019 Dec 26;14(12):e0221745. doi: 10.1371/journal.pone.0221745 (PMC6932809; doi:10.1371/journal.pone.0221745)
Supplement: S1 Table — (PDF) [file pone.0221745.s001.pdf]

Time point	Pathway			Significantly changed metabolites involved in the pathway						
24 hpi	Glyoxylate and dicarboxylate metabolism			Citrate	Formate	Glycerate	Glycolate	Isocitrate	Malate	Oxalacetate
		SAI	Mean	0.86102	0.00043	0.00216	0.00090	0.86257	0.02338	0.00236
			Std	0.02227	0.00015	0.00028	0.00029	0.02620	0.00758	0.00092
		CHH DSI	Mean	0.95117	0.00011	0.00029	0.00011	0.96713	0.00586	0.00405
			Std	0.00534	0.00003	0.00017	0.00006	0.00372	0.00088	0.00092
		p Value		0.00333	0.00262	0.00001	0.00097	0.00387	0.00647	0.02317
	Nicotinate and nicotinamide metabolism			Deamino-NAD$^+$	NAD$^+$	NADP$^+$	Niacinamide	Nicotinate		
		SAI	Mean	0.00649	0.01047	0.00855	0.00765	0.00683		
			Std	0.00309	0.00385	0.00321	0.00272	0.00262		
		CHH DSI	Mean	0.00115	0.00185	0.00144	0.00162	0.00170		
			Std	0.00010	0.00013	0.00010	0.00057	0.00050		
		p Value		0.00819	0.00273	0.00288	0.00255	0.00449		
	Alanine, aspartate and glutamate metabolism			Alanine	Asparagine	Aspartate	Glutamate	Glutamine	Oxalacetate	Pyruvate
		SAI	Mean	0.02569	1.71243	0.01715	0.05132	0.05291	0.00236	0.00110
			Std	0.01034	0.03783	0.00299	0.02001	0.02437	0.00092	0.00082
		CHH DSI	Mean	0.00122	1.89366	0.00858	0.00514	0.00523	0.00405	0.00014
			Std	0.00032	0.01192	0.00099	0.00216	0.00201	0.00092	0.00008
		p Value		0.00612	0.00026	0.00221	0.00652	0.01178	0.02317	0.03410
	Pyruvate metabolism			Acetaldehyde	Acetate	Lactate	Malate	Oxalacetate	Propylene glycol	Pyruvate
		SAI	Mean	0.00339	0.01676	0.00360	0.02338	0.00236	0.01738	0.00110
			Std	0.00169	0.01120	0.00143	0.00758	0.00092	0.00864	0.00082
		CHH DSI	Mean	0.00037	0.00148	0.00040	0.00586	0.00405	0.00817	0.00014
			Std	0.00009	0.00065	0.00012	0.00088	0.00092	0.00013	0.00008
		p Value		0.00714	0.02045	0.00266	0.00647	0.02317	0.04766	0.03410
	Nitrogen metabolism			Cystathionine	Glutamate	Glutamine				
		SAI	Mean	1.64975	0.05132	0.05291				
			Std	0.17732	0.02001	0.02437				
		CHH DSI	Mean	1.92118	0.00514	0.00523				
			Std	0.00805	0.00216	0.00201				
		p Value		0.01327	0.00652	0.01178				
48 hpi	Nicotinate and nicotinamide metabolism			Deamino-NAD$^+$	NAD$^+$	NADP$^+$	Niacinamide	Nicotinate		
		SAI	Mean	0.00603	0.00947	0.00763	0.00701	0.00638		
			Std	0.00392	0.00600	0.00479	0.00448	0.00391		
		CHH DSI	Mean	0.00107	0.00171	0.00134	0.00139	0.00147		
			Std	0.00020	0.00026	0.00027	0.00075	0.00068		
		p Value		0.02665	0.02478	0.02361	0.02737	0.02707		

SAI: saline-injected group; black bar, CHH DSI: CHH double-stranded RNA-injected group. Data are presented as a mean ± standard deviation (std) (n = 6). Those metabolites whose concentration significantly increased after CHH dsRNA treatment were underlined, while those of the rest of the listed metabolites significantly decreased. 24 and 48hpi: 24 and 48 hour post injection, respectively. Statistically significant value for Student's t-test (p Value) was given.
