# Supplementary material for: Regulation of amino acid and nucleotide metabolism by crustacean hyperglycemic hormone in the muscle and hepatopancreas of the crayfish Procambarus clarkia
Source: PLoS One. 2019 Dec 26;14(12):e0221745. doi: 10.1371/journal.pone.0221745 (PMC6932809; doi:10.1371/journal.pone.0221745)
Supplement: S2 Table — (PDF) [file pone.0221745.s002.pdf]

Time point	Pathway			Significantly changed metabolites involved in the pathway		
24 hpi	Pyruvate metabolism			Acetaldehyde	Acetate	Pyruvate
		SAI	Mean	0.00131	0.00498	0.00085
			Std	0.00050	0.00070	0.00020
		CHH DSI	Mean	0.00079	0.00412	0.00062
			Std	0.00023	0.00059	0.00017
		p Value		0.02157	0.02756	0.03826
	Glycolysis or Gluconeogenesis			Acetaldehyde	Acetate	Pyruvate
		SAI	Mean	0.00131	0.00498	0.00085
			Std	0.00050	0.00070	0.00020
		CHH DSI	Mean	0.00079	0.00412	0.00062
			Std	0.00023	0.00059	0.00017
		p Value		0.02157	0.02756	0.03826
48 hpi	Glycine, serine and threonine metabolism			2-Oxobutyrate	Sarcosine	
		SAI	Mean	0.00364	0.00310	
			Std	0.00053	0.00062	
		CHH DSI	Mean	0.00294	0.00214	
			Std	0.00058	0.00023	
		p Value		0.04364	0.01102	

Abbreviations are as those indicated in Supplementary Table 1.
