# Supplementary material for: Regulation of amino acid and nucleotide metabolism by crustacean hyperglycemic hormone in the muscle and hepatopancreas of the crayfish Procambarus clarkia
Source: PLoS One. 2019 Dec 26;14(12):e0221745. doi: 10.1371/journal.pone.0221745 (PMC6932809; doi:10.1371/journal.pone.0221745)
Supplement: S3 Table — (PDF) [file pone.0221745.s003.pdf]

Time point	Metabolite set			Significantly changed metabolites involved in the metabolite set								
	Ammonia Recycling			ADP	AMP	Asparagine	Aspartate	ATP	Glutamate	Glutamine		
		SAI	Mean	0.00368	0.00464	1.71243	0.01715	0.00361	0.05132	0.05291		
			Std	0.00126	0.00136	0.03783	0.00299	0.00127	0.02001	0.02437		
		CHH DSI	Mean	0.00055	0.00067	1.89366	0.00858	0.00059	0.00514	0.00523		
			Std	0.00005	0.00015	0.01192	0.00099	0.00005	0.00216	0.00201		
		p Value		0.00173	0.00079	0.00026	0.00221	0.00213	0.00652	0.01178		
				Histidine	NAD+	NADH	Pyruvate	Serine	Urocanate			
		SAI	Mean	0.85207	0.01047	0.01958	0.00110	0.00411	0.00705			
			Std	0.03509	0.00385	0.00537	0.00082	0.00117	0.00193			
		CHH DSI	Mean	0.97869	0.00185	0.00729	0.00014	0.00056	0.00151			
			Std	0.00290	0.00013	0.00076	0.00008	0.00035	0.00059			
		p Value		0.00539	0.00273	0.00658	0.03410	0.00189	0.00061			
	Nicotinate and Nicotinamide Metabolism			1-Methylnicotinamide	ADP	AMP	ATP	Deamino-NAD+	Glutamate	Glutamine		
		SAI	Mean	0.00218	0.00368	0.00464	0.00361	0.00649	0.05132	0.05291		
			Std	0.00088	0.00126	0.00136	0.00127	0.00309	0.02001	0.02437		
		CHH DSI	Mean	0.00054	0.00055	0.00067	0.00059	0.00115	0.00514	0.00523		
			Std	0.00015	0.00005	0.00015	0.00005	0.00010	0.00216	0.00201		
		p Value		0.00560	0.00173	0.00079	0.00213	0.00819	0.00652	0.01178		
				NAD+	NADH	NADP+	NADPH	Niacinamide	Nicotinate	Quinolinate		
		SAI	Mean	0.01047	0.01958	0.00855	0.01487	0.00765	0.00683	0.00488		
			Std	0.00385	0.00537	0.00321	0.00684	0.00272	0.00262	0.00200		
		CHH DSI	Mean	0.00185	0.00729	0.00144	0.00406	0.00162	0.00170	0.00105		
			Std	0.00013	0.00076	0.00010	0.00046	0.00057	0.00050	0.00037		
		p Value		0.00273	0.00658	0.00288	0.01166	0.00255	0.00449	0.00509		
	Aspartate Metabolism			Acetate	AMP	Arginine	Asparagine	Aspartate	ATP			
		SAI	Mean	0.01676	0.00464	0.10689	1.71243	0.01715	0.00361			
			Std	0.01120	0.00136	0.02768	0.03783	0.00299	0.00127			
		CHH DSI	Mean	0.00148	0.00067	0.01707	1.89366	0.00858	0.00059			
			Std	0.00065	0.00015	0.00296	0.01192	0.00099	0.00005			
		p Value		0.02045	0.00079	0.00184	0.00026	0.00221	0.00213			
				Citrulline	Glutamate	Glutamine	GTP	Malonate	Oxalacetate			
		SAI	Mean	0.96721	0.05132	0.05291	0.00256	0.26767	0.00236			
			Std	0.73536	0.02001	0.02437	0.00099	0.40341	0.00092			
		CHH DSI	Mean	1.89610	0.00514	0.00523	0.00036	0.77390	0.00405			
			Std	0.00827	0.00216	0.00201	0.00008	0.00947	0.00092			
		p Value		0.02703	0.00652	0.01178	0.00274	0.02765	0.02317			
	Pyruvate metabolism			Acetaldehyde	Acetate	ADP	AMP	ATP	GTP	Lactate		
		SAI	Mean	0.00339	0.01676	0.00368	0.00464	0.00361	0.00256	0.00360		
			Std	0.00169	0.01120	0.00126	0.00136	0.00127	0.00099	0.00143		
		CHH DSI	Mean	0.00037	0.00148	0.00055	0.00067	0.00059	0.00036	0.00040		
			Std	0.00009	0.00065	0.00005	0.00015	0.00005	0.00008	0.00012		
		p Value		0.00714	0.02045	0.00173	0.00079	0.00213	0.00274	0.00266		
				NAD+	NADH	NADP+	NADPH	Oxalacetate	Propylene glycol	Pyruvate		
		SAI	Mean	0.01047	0.01958	0.00855	0.01487	0.00236	0.01738	0.00110		
			Std	0.00385	0.00537	0.00321	0.00684	0.00092	0.00864	0.00082		
		CHH DSI	Mean	0.00185	0.00729	0.00144	0.00406	0.00405	0.00817	0.00014		
			Std	0.00013	0.00076	0.00010	0.00046	0.00092	0.00013	0.00008		
		p Value		0.00273	0.00658	0.00288	0.01166	0.02317	0.04766	0.03410		
	Alanine Metabolism			Alanine	ADP	AMP	ATP	Glutamate	Oxalacetate	Pyruvate		
		SAI	Mean	0.02569	0.00368	0.00464	0.00361	0.05132	0.00236	0.00110		
			Std	0.01034	0.00126	0.00136	0.00127	0.02001	0.00092	0.00082		
		CHH DSI	Mean	0.00122	0.00055	0.00067	0.00059	0.00514	0.00405	0.00014		
			Std	0.00032	0.00005	0.00015	0.00005	0.00216	0.00092	0.00008		
		p Value		0.00612	0.00173	0.00079	0.00213	0.00652	0.02317	0.03410		
	Glutamate Metabolism			Alanine	ADP	AMP	Aspartate	ATP	Deamino-NAD+	Glutamate		
		SAI	Mean	0.02569	0.00368	0.00464	0.01715	0.00361	0.00649	0.05132		
			Std	0.01034	0.00126	0.00136	0.00299	0.00127	0.00309	0.02001		
		CHH DSI	Mean	0.00122	0.00055	0.00067	0.00858	0.00059	0.00115	0.00514		
			Std	0.00032	0.00005	0.00015	0.00099	0.00005	0.00010	0.00216		
		p Value		0.00612	0.00173	0.00079	0.00221	0.00213	0.00819	0.00652		
				Glutamine	NAD+	NADH	NADP+	NADPH	Oxalacetate	Pyruvate		
		SAI	Mean	0.05291	0.01047	0.01958	0.00855	0.01487	0.00236	0.00110		
			Std	0.02437	0.00385	0.00537	0.00321	0.00684	0.00092	0.00082		
		CHH DSI	Mean	0.00523	0.00185	0.00729	0.00144	0.00406	0.00405	0.00014		
			Std	0.00201	0.00013	0.00076	0.00010	0.00046	0.00092	0.00008		
		p Value		0.01178	0.00273	0.00658	0.00288	0.01166	0.02317	0.03410		
	Purine Metabolism			Adenine	Adenosine	ADP	AMP	Aspartate	ATP	Glutamate	Glutamine	GTP
		SAI	Mean	0.00298	0.00478	0.00368	0.00464	0.01715	0.00361	0.05132	0.05291	0.00256
			Std	0.00108	0.00132	0.00126	0.00136	0.00299	0.00127	0.02001	0.02437	0.00099
		CHH DSI	Mean	0.00065	0.00067	0.00055	0.00067	0.00858	0.00059	0.00514	0.00523	0.00036
			Std	0.00021	0.00007	0.00005	0.00015	0.00099	0.00005	0.00216	0.00201	0.00008
		p Value		0.00297	0.00060	0.00173	0.00079	0.00221	0.00213	0.00652	0.01178	0.00274
				Guanosine	Hypoxanthine	Inosine	NAD+	NADH	NADP+	NADPH	Xanthine	Xanthosine
		SAI	Mean	0.00253	0.00116	0.00804	0.01047	0.01958	0.00855	0.01487	0.00151	0.00588
			Std	0.00095	0.00041	0.00437	0.00385	0.00537	0.00321	0.00684	0.00066	0.00135
		CHH DSI	Mean	0.00032	0.00025	0.00089	0.00185	0.00729	0.00144	0.00406	0.00026	0.00073
			Std	0.00003	0.00008	0.00014	0.00013	0.00076	0.00010	0.00046	0.00011	0.00024
		p Value		0.00233	0.00241	0.01020	0.00273	0.00658	0.00288	0.01166	0.00557	0.00089
	Arginine and Proline Metabolism			ADP	AMP	Arginine	Aspartate	ATP	Citrulline	Glutamate		
		SAI	Mean	0.00368	0.00464	0.10689	0.01715	0.00361	0.96721	0.05132		
			Std	0.00126	0.00136	0.02768	0.00299	0.00127	0.73536	0.02001		
		CHH DSI	Mean	0.00055	0.00067	0.01707	0.00858	0.00059	1.89610	0.00514		
			Std	0.00005	0.00015	0.00296	0.00099	0.00005	0.00827	0.00216		
		p Value		0.00173	0.00079	0.00184	0.00221	0.00213	0.02703	0.00652		
				NAD+	NADH	NADP+	NADPH	Oxalacetate	Proline	Urea		
		SAI	Mean	0.01047	0.01958	0.00855	0.01487	0.00236	0.05553	0.00095		
			Std	0.00385	0.00537	0.00321	0.00684	0.00092	0.02934	0.00043		
		CHH DSI	Mean	0.00185	0.00729	0.00144	0.00406	0.00405	0.00660	0.00008		
			Std	0.00013	0.00076	0.00010	0.00046	0.00092	0.00237	0.00006		
		p Value		0.00273	0.00658	0.00288	0.01166	0.02317	0.00940	0.00412		
	Glycine and Serine Metabolism			Alanine	ADP	AMP	Arginine	ATP	Cystathionine	Glutamate	Glycerate	
		SAI	Mean	0.02569	0.00368	0.00464	0.10689	0.00361	1.64975	0.05132	0.00216	
			Std	0.01034	0.00126	0.00136	0.02768	0.00127	0.17732	0.02001	0.00028	
		CHH DSI	Mean	0.00122	0.00055	0.00067	0.01707	0.00059	1.92118	0.00514	0.00029	
			Std	0.00032	0.00005	0.00015	0.00296	0.00005	0.00805	0.00216	0.00017	
		p Value		0.00612	0.00173	0.00079	0.00184	0.00213	0.01327	0.00652	0.00001	
				Methionine	NAD+	NADH	O-Phosphoserine	Pyruvate	Serine	α-Ketobutyrate		
		SAI	Mean	0.04774	0.01047	0.01958	0.00680	0.00110	0.00411	0.00366		
			Std	0.01984	0.00385	0.00537	0.00102	0.00082	0.00117	0.00084		
		CHH DSI	Mean	0.00524	0.00185	0.00729	0.00116	0.00014	0.00056	0.00157		
			Std	0.00198	0.00013	0.00076	0.00053	0.00008	0.00035	0.00022		
		p Value		0.02306	0.00273	0.00658	0.00000	0.03410	0.00189	0.00442		

	Warburg Effect			ADP	ATP	Citrate	Glutamate	Glutamine	GTP	Isocitrate		
		SAI	Mean	0.00368	0.00361	0.86102	0.05132	0.05291	0.00256	0.86257		
			Std	0.00126	0.00127	0.02227	0.02001	0.02437	0.00099	0.02620		
		CHH DSI	Mean	0.00055	0.00059	0.95117	0.00514	0.00523	0.00036	0.96713		
			Std	0.00005	0.00005	0.00534	0.00216	0.00201	0.00008	0.00372		
		p Value		0.00173	0.00213	0.00333	0.00652	0.01178	0.00274	0.00387		
				Lactate	NAD+	NADH	NADP+	NADPH	Oxalacetate	Pyruvate		
		SAI	Mean	0.00360	0.01047	0.01958	0.00855	0.01487	0.00236	0.00110		
			Std	0.00143	0.00385	0.00537	0.00321	0.00684	0.00092	0.00082		
		CHH DSI	Mean	0.00040	0.00185	0.00729	0.00144	0.00406	0.00405	0.00014		
			Std	0.00012	0.00013	0.00076	0.00010	0.00046	0.00092	0.00008		
		p Value		0.00266	0.00273	0.00658	0.00288	0.01166	0.02317	0.03410		
	Citric Acid Cycle			ADP	ATP	Citrate	GTP	Isocitrate				
		SAI	Mean	0.00368	0.00361	0.86102	0.00256	0.86257				
			Std	0.00126	0.00127	0.02227	0.00099	0.02620				
		CHH DSI	Mean	0.00055	0.00059	0.95117	0.00036	0.96713				
			Std	0.00005	0.00005	0.00534	0.00008	0.00372				
		p Value		0.00173	0.00213	0.00333	0.00274	0.00387				
		Metabolites		NAD+	NADH	Oxalacetate	Pyruvate					
		SAI	Mean	0.01047	0.01958	0.00236	0.00110					
			Std	0.00385	0.00537	0.00092	0.00082					
		CHH DSI	Mean	0.00185	0.00729	0.00405	0.00014					
			Std	0.00013	0.00076	0.00092	0.00008					
		p Value		0.00273	0.00658	0.02317	0.03410					
	Nicotinate and nicotinamide metabolism			1-Methylnicotinamide	ADP	AMP	ATP	Deamino-NAD$^+$	Glutamate	Glutamine		
		SAI	Mean	0.00198	0.00317	0.00383	0.00312	0.00603	0.03291	0.03418		
			Std	0.00122	0.00201	0.00235	0.00199	0.00392	0.01953	0.02023		
		CHH DSI	Mean	0.00047	0.00051	0.00061	0.00054	0.00107	0.00745	0.00716		
			Std	0.00021	0.00014	0.00021	0.00014	0.00020	0.00377	0.00337		
		p Value		0.02884	0.02250	0.02015	0.02443	0.02665	0.02358	0.02169		
				NAD$^+$	NADH	NADP$^+$	Niacinamide	Nicotinate	Quinolinate	S-Adenosylhomocysteine		
		SAI	Mean	0.00947	0.02090	0.00763	0.00701	0.00638	0.00461	1.86813		
			Std	0.00600	0.00148	0.00479	0.00448	0.00391	0.00295	0.05542		
		CHH DSI	Mean	0.00171	0.01119	0.00134	0.00139	0.00147	0.00090	1.93546		
			Std	0.00026	0.01075	0.00027	0.00075	0.00068	0.00050	0.00596		
		p Value		0.02478	0.03828	0.02361	0.02737	0.02707	0.02701	0.03075		
48 hpi	Glycine and Serine Metabolism			ADP	Alanine	AMP	Arginine	ATP	Betaine	Cystathionine	Cysteine	Glutamate
		SAI	Mean	0.00317	0.01422	0.00383	0.07059	0.00312	0.00048	1.82464	1.79923	0.03291
			Std	0.00201	0.01106	0.00235	0.04658	0.00199	0.00022	0.07725	0.08638	0.01953
		CHH DSI	Mean	0.00051	0.00141	0.00061	0.02151	0.00054	0.00128	1.90972	1.89782	0.00745
			Std	0.00014	0.00086	0.00021	0.00928	0.00014	0.00093	0.02842	0.02617	0.00377
		p Value		0.02250	0.03630	0.02015	0.04903	0.02443	0.04693	0.04219	0.03728	0.02358
				Glycerate	Guanidoacetate	Methionine	NAD$^+$	NADH	Ornithine	O-Phosphoserine	α-Ketobutyrate	S-Adenosylhomocysteine
		SAI	Mean	0.00170	0.00046	0.02819	0.00947	0.02090	0.98384	0.00556	0.00270	1.86813
			Std	0.00098	0.00027	0.01556	0.00600	0.00148	0.02349	0.00307	0.00087	0.05542
		CHH DSI	Mean	0.00037	0.00011	0.00775	0.00171	0.01119	0.95700	0.00102	0.00163	1.93546
			Std	0.00027	0.00010	0.00438	0.00026	0.01075	0.01798	0.00079	0.00030	0.00596
		p Value		0.01972	0.02131	0.02242	0.02478	0.03828	0.04424	0.01410	0.02750	0.03075
	Ammonia Recycling			ADP	AMP	Asparagine	ATP	Biotin	Glutamate			
		SAI	Mean	0.00317	0.00383	1.80641	0.00312	0.97497	0.03291			
			Std	0.00201	0.00235	0.07290	0.00199	0.02394	0.01953			
		CHH DSI	Mean	0.00051	0.00061	1.89172	0.00054	0.93995	0.00745			
			Std	0.00014	0.00021	0.01105	0.00014	0.01484	0.00377			
		p Value		0.02250	0.02015	0.03480	0.02443	0.01380	0.02358			
				Glutamine	Histidine	NAD$^+$	NADH	Serine	Urocanate			
		SAI	Mean	0.03418	0.91398	0.00947	0.02090	0.00349	0.00613			
			Std	0.02023	0.04838	0.00600	0.00148	0.00203	0.00390			
		CHH DSI	Mean	0.00716	0.97524	0.00171	0.01119	0.00076	0.00130			
			Std	0.00337	0.01151	0.00026	0.01075	0.00063	0.00077			
		p Value		0.02169	0.02596	0.02478	0.03828	0.02045	0.02844			

Abbreviations are as those indicated in Supplementary Table 1.
