# Supplementary material for: Regulation of amino acid and nucleotide metabolism by crustacean hyperglycemic hormone in the muscle and hepatopancreas of the crayfish Procambarus clarkia
Source: PLoS One. 2019 Dec 26;14(12):e0221745. doi: 10.1371/journal.pone.0221745 (PMC6932809; doi:10.1371/journal.pone.0221745)
Supplement: S4 Table — (PDF) [file pone.0221745.s004.pdf]

Time point	Metabolite set			Significantly changed metabolites involved in the metabolite set		
				**Acetate**	**Butyrate**	**Caprate**
**24 hpi**	**Fatty Acid Biosynthesis**	**SAI**	**Mean**	**0.00498**	**0.00767**	**0.01121**
			**Std**	**0.00070**	**0.00196**	**0.00149**
		**CHH DSI**	**Mean**	**0.00412**	**0.00500**	**0.00907**
			**Std**	**0.00059**	**0.00079**	**0.00173**
		**p Value**		**0.02756**	**0.00417**	**0.03196**

Abbreviations are as those indicated in Supplementary Table 1.
